# Retraction: Recurrent Radiculopathy Caused by an Epidural Cervical Plasmacytoma Revealing Plasma Cell Leukemia: A Case Report and Literature Review

**DOI:** 10.7759/cureus.r249

**Published:** 2026-09-24

**Authors:** Luis Garibay, Jose-Carlos Sauri-Barraza, Eduardo Callejas Ponce, Jesus E Valdez-Aguilar, Ulises García-González, Jorge D. Pérez Ruiz, Carlo Enrico Bañuelos Aluzzi, Miguel A Rodriguez

**Affiliations:** 1 Spine Surgery, Centro Médico ABC, Mexico City, MEX; 2 Spine Surgery, Axis Premium Spine Care, Mexico City, MEX; 3 Spine Clinic, Centro Médico ABC, Mexico City, MEX; 4 Neurosurgery, Hospital Central Sur de Alta Especialidad "PEMEX", Mexico City, MEX; 5 Neurosurgery, Centro Médico ABC, Mexico City, MEX

This article has been retracted by the Editor-in-Chief due to concerns over duplicate publication and lack of co-author and patient consent. This article describes the same patient and clinical case as a manuscript by a different, partially overlapping author group that was under review at Surgical Neurology International when this article was submitted, and was accepted by that journal on July 22, 2026. The authors agree with the decision to retract.

